# Cross-talk and regulation between glutamate and GABA_B_ receptors

**DOI:** 10.3389/fncel.2015.00135

**Published:** 2015-04-10

**Authors:** Sriharsha Kantamneni

**Affiliations:** Bradford School of Pharmacy, School of Life Sciences, University of BradfordBradford, West Yorkshire, UK

**Keywords:** glutamate receptor, NMDAR, GABA_B_R, AMPAR, AKAP, receptor regulation, receptor trafficking and mGluR

## Abstract

Brain function depends on co-ordinated transmission of signals from both excitatory and inhibitory neurotransmitters acting upon target neurons. NMDA, AMPA and mGluR receptors are the major subclasses of glutamate receptors that are involved in excitatory transmission at synapses, mechanisms of activity dependent synaptic plasticity, brain development and many neurological diseases. In addition to canonical role of regulating presynaptic release and activating postsynaptic potassium channels, GABA_B_ receptors also regulate glutamate receptors. There is increasing evidence that metabotropic GABA_B_ receptors are now known to play an important role in modulating the excitability of circuits throughout the brain by directly influencing different types of postsynaptic glutamate receptors. Specifically, GABA_B_ receptors affect the expression, activity and signaling of glutamate receptors under physiological and pathological conditions. Conversely, NMDA receptor activity differentially regulates GABA_B_ receptor subunit expression, signaling and function. In this review I will describe how GABA_B_ receptor activity influence glutamate receptor function and* vice versa*. Such a modulation has widespread implications for the control of neurotransmission, calcium-dependent neuronal function, pain pathways and in various psychiatric and neurodegenerative diseases.

## Introduction

Most excitatory signals that a neuron receives are mediated via glutamate receptors whereas most inhibitory signals are mediated via γ-aminobutyric acid (GABA) receptors (Cherubini et al., 1991; Hollmann and Heinemann, 1994). Many factors influence the regulation of excitatory and inhibitory synaptic inputs on a given neuron. One important factor is the subtype of neurotransmitter receptors present at not only the correct location to receive the appropriate signals but also their abundance at synapses (Dingledine et al., 1999; Sheng and Kim, 2011). Thus the molecular mechanisms that regulate receptor expression and localization at specific sites are of considerable importance. This review will describe the recent advances in our understanding of the molecular mechanisms underlying glutamate and GABA_B_ receptors cross-talk and discuss the roles of specific proteins that might control these processes.

Glutamate receptors are the major excitatory neurotransmitter receptors in the brain and play an important role in neural plasticity and development. Improper function of glutamate receptors is involved in various psychiatric and neurodegenerative diseases (Mattson, 2008; Musazzi et al., 2013). N-methyl-D-aspartate receptors (NMDARs), α-amino-3-hydroxy-5-methyl-4-isoxazolepropionate receptors (AMPARs) and kainate receptors are glutamate-gated ion channels, whereas metabotropic glutamate receptors (mGluRs) are G-protein-coupled receptors (GPCRs) that signal downstream via interaction with heterotrimeric G-proteins. Pharmacological and molecular biological studies have revealed that glutamate receptors exist as different subclasses, where receptor subtypes comprise multiple subunits such as NMDA receptors (GluN1 to GluN3), AMPA receptors (GluA1 to GluA4), kainate receptors (GluK1 to GluK5) and mGlu receptors (mGluR1 to mGluR8) (for reviews, see Nakanishi et al., 1998; Lodge, 2009; Nicoletti et al., 2011).

Conversely, GABA receptors are the primary proteins responsible for inhibitory responses in the brain. Metabotropic GABA receptors (GABA_B_Rs) are GPCRs that can mediate slow inhibitory neurotransmission in the CNS. GABA_B_Rs are located at both presynaptic and postsynaptic compartments and changes in their number, localization and activity affect the level of synaptic inhibition. Presynaptic GABA_B_Rs inhibit release of neurotransmitter by inhibiting Ca^2+^ channels (Wu and Saggau, 1995; Takahashi et al., 1998). Activation of postsynaptic GABA_B_Rs activates inwardly rectifying K^+^ channels (GIRK) to generate slow inhibitory postsynaptic potentials (reviewed in Marshall et al., 1999; Bowery et al., 2002; Gainetdinov et al., 2004). The GABA_B_R is a heteromeric GPCR consisting of GABA_B1_ and GABA_B2_ subunits that exert much longer lasting synaptic inhibition compared to GABA_A_ ion channels (Marshall et al., 1999; Watanabe et al., 2002). The ligand-binding domain (Malitschek et al., 1999) is present in GABA_B1_ subunit and G-proteins interact with GABA_B2_ to regulate adenylate cyclase, GIRK channels and Ca^2+^ channels (Robbins et al., 2001). A large body of work over the last 20 years has demonstrated that GABA_B_ receptors are regulated via mechanisms distinct from those utilized by many classical GPCRs such as the β_2_-adrenergic receptor (Bettler and Tiao, 2006). For example, following agonist exposure most GPCRs are phosphorylated and endocytosed from the cell surface into intracellular compartments and then either down-regulated via lysosomal or proteasomal degradation or recycled back to the cell surface following agonist removal. In contrast, cell surface GABA_B_ receptor levels are not significantly altered upon receptor stimulation in cultured cortical and hippocampal neurons (Fairfax et al., 2004; Bettler and Tiao, 2006). GABA_B_ receptors are very stable at the plasma membrane even after agonist exposure with little internalization in cultured neurons. The absence of receptor endocytosis correlates with lack of arrestin recruitment and agonist-induced phosphorylation (Fairfax et al., 2004). Surprisingly, increased phosphorylation at serine 892 in GABA_B2_ subunit decreased degradation rates and stabilizes surface GABA_B_Rs in neurons (Couve et al., 2004; Fairfax et al., 2004).

The main regulatory sites on both glutamate receptors and GABA_B_Rs are their intracellular C-terminal tails. Depending on the activity or stimulation received by the receptors, the C-terminal domains bind to various proteins including enzymes, scaffolds, and trafficking and signaling proteins (De La Rue and Henley, 2002). These sites sometimes also mediate complex formation during a cross-talk between the receptors. Many immunocytochemical and electron microscopy studies have demonstrated that glutamatergic synapses are enriched with GABA_B_Rs (Fritschy et al., 1999; Luján and Shigemoto, 2006). There is also increasing evidence that NMDARs, AMPARs and mGluRs are modulated directly and sometimes indirectly by GABA_B_Rs (Morrisett et al., 1991; Hirono et al., 2001; Otmakhova and Lisman, 2004; Tabata et al., 2004; Sun et al., 2006; Chalifoux and Carter, 2010; Gandal et al., 2012; Terunuma et al., 2014). Conversely, GABA_B_R subunits are differentially regulated by glutamate receptor subtypes under various stimulation protocols (Vargas et al., 2008; Cimarosti et al., 2009; Guetg et al., 2010; Maier et al., 2010; Terunuma et al., 2010; Kantamneni et al., 2014). The sections below in this review will follow this theme of regulation or modulation between GABA_B_ and glutamate receptors. This cross-talk provides important regulatory mechanisms, for example, in altering presynaptic release or changes to membrane potential, but also alters the function of glutamate receptors, which may prove useful in a therapeutic context.

## GABA_B_R-Mediated Regulation of Glutamate Receptor Function

### GABA_B_R Regulation of NMDAR-Dependent Post-Synaptic Calcium Signals

The major synaptic Ca^2+^ signals in the brain are mediated via NMDARs, which are crucial for activity-dependent changes in synaptic plasticity (Bliss and Collingridge, 1993; Mainen et al., 1999; Malenka and Bear, 2004). These Ca^2+^ signals are thought to be inhibited by GABA_B_ receptors via modulation of K^+^ channels, resulting in a hyperpolarization that decreases the Ca^2+^ influx and overall current by enhancing Mg^2+^ blockade of NMDARs. (Morrisett et al., 1991; Otmakhova and Lisman, 2004; Deng et al., 2009). Interestingly, it has also been demonstrated recently that Ca^2+^ influx via NMDARs is inhibited by GABA_B_ receptor activation (Chalifoux and Carter, 2010). This effect on NMDARs is independent of K^+^ channel and voltage sensitive Ca^2+^ channel activation, Gβγ subunits and internal Ca^2+^ stores. Via coupling to Gα_i_/Gα_o_ G proteins, GABA_B_Rs inhibit adenylate cyclase to reduce PKA activity by decreasing cAMP levels. The Ca^2+^ influx via NMDA receptors is normally increased by PKA activity and reduction of PKA activity by GABA_B_Rs inhibits Ca^2+^ signals (Chalifoux and Carter, 2010). GABA_B_R-mediated postsynaptic modulation through the PKA pathway does not affect synaptic currents mediated by NMDA or AMPA receptors (Chalifoux and Carter, 2010). As outlined below, protein kinases such as PKA and phosphatases such as PP1/2 and calcineurin (CaN) are regulated via AKAPs (A Kinase Anchoring Proteins) and mediate signaling where they act as scaffold molecules (see below for further insights).

### NMDAR and GABA_B_R Cross-Talk in Disease

Recently it has been demonstrated that, there is clear interplay between GABA_B_ and NMDA receptors not only in physiological functions but also in pathological situations. Altered NMDAR activity is observed in models of pain and neuropsychiatric disorders, but an interesting phenomenon is that these phenotypes can be rescued with GABA_B_R ligands. For example, in diabetic neuropathy, NMDAR expression is increased in spinal cord dorsal horn, while GABA_B_ receptors are down regulated at protein level (Wang et al., 2011). Using streptozotocin (STZ)-induced diabetic neuropathy rat models (STZ), it has been found that intrathecal injection of the GABA_B_R agonist baclofen significantly increased paw withdrawal threshold. This effect was blocked with pre-treatment of CGP55845, a GABA_B_R—selective antagonist (Bai et al., 2014). In STZ rats, changes in expression were observed in both cyclic AMP response element-binding protein (CREB) and GluN2B, which were significantly increased at the protein (CREB and GluN2B) and mRNA level (GluN2B) in spinal cord. The higher expression levels of both GluN2B and phosphorylated CREB proteins were significantly reduced by administration of baclofen (Liu et al., 2014). Importantly, baclofen-induced reduction of GluN2B and CREB expression was abolished when CGP55845 was pre-administered, suggesting that GABA_B_R activation in the spinal cord dorsal horn can normalize NMDAR expression levels in diabetic neuropathic pain (Wang et al., 2011; Bai et al., 2014; Liu et al., 2014).

In contrast, reduced NMDA receptor functionality has been observed in neuropsychiatric disorders like intellectual disability, autism and schizophrenia (Gonda, 2012). For example, a mouse model expressing a reduced amount of GluN1 subunit (NR1^neo−/−^ mice) was characterized to mimic schizophrenic-like behavior (Mohn et al., 1999). These mice have increased power in the gamma (30–80 Hz) EEG range during rest, but show a reduced auditory-stimulus evoked gamma power (reduced gamma signal-to-noise), causing changes in excitatory/inhibitory balance, and express treatment resistant symptoms of autism and schizophrenia (Gandal et al., 2012). Treating NR1^neo−/−^ mice with baclofen restored excitatory/inhibitory balance, neural synchrony and also improved social function and spatial memory deficits (Gandal et al., 2012). To summarize, diseases characterized by NMDA receptor dysfunction, have the additional possibility of using GABA_B_ receptors as an appropriate target for therapy that could possibly pave the way to restore abnormalities in many other neurological diseases.

### GABA_B_R Cross-Talk with AMPARs

Surface expression of AMPA receptors was increased in a knock-in mouse model in which wild-type GABA_B2_R was replaced with a S783A-mutated version which cannot be phosphorylated (Terunuma et al., 2014). The S783 on GABA_B2_ subunit is phosphorylated by AMP-dependent protein kinase (AMPK), which in-turn enhances receptor coupling to GIRKs (Kuramoto et al., 2007). Activating NMDARs transiently results in increased phosphorylation whereas prolonged activation results in dephosphorylation of GABA_B_Rs by protein phosphatase 2A (PP2A). GABA_B_Rs stability at cell surface is due to high constitutive phosphorylation of GABA_B2_R and dephosphorylation of this subunit selectively targets the receptors for lysosomal degradation (Fairfax et al., 2004; Terunuma et al., 2010). The expression of GABA_B_R was increased with the mutation due to reduced degradation, leading to decreased level of Arc/Arg3.1 protein necessary for memory consolidation. This, in turn, increased the number of excitatory synapses, PSD95 protein expression and cell surface AMPA receptors. This cross-talk demonstrates a crucial role for GABA_B_Rs in regulating excitatory synaptic transmission and neuronal architecture (Terunuma et al., 2014).

### GABA_B_R Cross-Talk with mGluRs

Long-term depression (LTD) at cerebellar parallel fiber Purkinje cell synapses is a form of synaptic plasticity critical for cerebellar motor learning and requires the activation of the metabotropic glutamate receptor mGluR1 (Ichise et al., 2000; Ito, 2001). GABA_B_Rs are concentrated at cerebellar parallel fiber Purkinje cell synapses and have many functions that are both dependent and independent of GABA. GABA_B_Rs and mGluR1 are highly co-expressed in cerebellar Purkinje cells, and display very similar subcellular localizations throughout development (Ige et al., 2000; Luján and Shigemoto, 2006; Rives et al., 2009). Electrophysiological studies have shown that at Purkinje cell synapses, GABA_B_R activation inhibits neurotransmitter release by inhibiting calcium channels as well as affecting release processes (Dittman and Regehr, 1996, 1997; Vigot and Batini, 1997). Extracellular Ca^2+^ interacts with GABA_B_R in cerebellar Purkinje cells, leading to an increase in the glutamate sensitivity of mGluR1. This sensitization of mGluR1 to glutamate is specifically mediated by GABA_B_Rs as it is absent in cells from GABA_B1_−/− animals. It has also been shown that both GPCRs form a complex in cerebellum and that extracellular Ca^2+^-mediated crosstalk is not mediated via G_i/o_ proteins (Tabata et al., 2004). Activity-dependent GABA_B_R inhibition by selective antagonists reduces the magnitude of LTD at parallel fiber Purkinje cell synapses (Kamikubo et al., 2007; Rives et al., 2009). In summary GABA_B_Rs not only mediate classical synaptic GABAergic neurotransmission but also regulate mGluR signaling and cerebellar synaptic plasticity.

## NMDAR-Mediated Regulation of GABA_B_R Function

GABA_B_Rs are very stable at cell surface in terms of agonist stimulation and the number of cell surface GABA_B_Rs is primarily controlled by glutamate and not GABA in central neurons (Fairfax et al., 2004; Vargas et al., 2008). Sustained application of glutamate leads to GABA_B_R endocytosis, trafficking to lysosomes and subsequent degradation, resulting in a decrease in receptor expression at the cell membrane (Vargas et al., 2008; Maier et al., 2010). Further dissection of the effect of glutamate indicated that activation of AMPA and NMDA receptors is required for the down-regulation of GABA_B_Rs and that this effect is enhanced by activation of the group I mGluRs (mGlu1 and mGlu5) (Maier et al., 2010). Activation of NMDARs alone leads to down-regulation and degradation of GABA_B1_ and GABA_B2_ subunits, thereby reducing cell surface expression (Guetg et al., 2010; Terunuma et al., 2010; Kantamneni et al., 2014). Mechanistically, NMDAR activation triggers GABA_B1_ subunit phosphorylation on Ser867 by CaMKII, causing a CaMKII-dependent down regulation (Guetg et al., 2010). In both hippocampal and cortical cultured neurons NMDAR activation also alters the phosphorylation state of GABA_B2_ subunit on Ser783, resulting in endocytosis and lysosomal degradation of the receptor complex (Terunuma et al., 2010). The GABA_B2_ subunit is also rapidly phosphorylated by AMPK upon NMDAR activation. Prolonged NMDAR activation subsequently results in GABA_B2_ subunit dephosphorylation by PP2A, which decreases the number of cell surface receptors (Terunuma et al., 2010).

Recently it has been shown that selective activation of synaptic NMDARs using chemically induced LTP (long-term potentiation) protocol (chem-LTP) leads to an increase in surface GABA_B_ receptors (Kantamneni et al., 2014). In the chem-LTP protocol, glycine (along with strychnine and bicuculline—to block glycine and GABA_A_ receptors, respectively) was used to specifically activate synaptic NMDARs, leading to significant increase in surface expression of AMPARs (Lu et al., 2001; Park et al., 2004). Prolonged activation of extrasynaptic NMDARs promotes cell death, whereas activation of synaptic NMDARs mediates synaptic plasticity and is thought to be involved in neuroprotection via modulation of nuclear Ca^2+^ signaling (Hardingham and Bading, 2010). Using the chem-LTP method, both GABA_B1_ and GABA_B2_ receptor subunit expression on the cell surface were increased in cultured rat hippocampal neurons due to enhanced receptor recycling from intracellular pools (Kantamneni et al., 2014).

GABA_B_R subunits are differentially regulated under oxygen/glucose deprivation (OGD) conditions, which stimulates release of excess glutamate resulting in excitotoxic activation of NMDARs (Papadia and Hardingham, 2007; Cimarosti et al., 2009; Kantamneni et al., 2014). After OGD, expression of GABA_B1_ subunits at the cell surface is increased via enhanced recycling, while total cellular and cell surface expression levels of GABA_B2_ subunits are decreased due to reduced recycling (Cimarosti et al., 2009; Kantamneni et al., 2014; Maier et al., 2014). Removing GABA_B2_ subunit will decrease the number of functional GABA_B_Rs, as both subunits are required for normal signaling. In conclusion, the above findings demonstrate that the expression and regulation of GABA_B_R subunits are dynamically regulated in response to synaptic and prolonged/global stimulation of NMDARs. Moreover, NMDAR regulation of GABA_B_Rs may be important under conditions of neurological disease, such as epilepsy or ischemia.

## Anchoring and Scaffold Proteins as Possible Mediators of GABA/Glutamate Receptor Cross-Talk

Both GABAergic and glutamatergic receptor complexes are regulated and orchestrated by anchoring and scaffold proteins, which are increasingly being implicated in the cross-talk between the two systems. Components of receptor signalosome are typically localized together via scaffold proteins, which co-assemble receptors with regulatory proteins such as protein kinases and phosphatases. AKAPs are typical examples of this class of scaffold proteins (Wong and Scott, 2004). For example, AKAP5 (or AKAP79/150) is thought to localize PKA, protein kinase C (PKC) and the calmodulin-activated protein phosphatase calcineurin (CaN) at specific synaptic sites to regulate excitatory synaptic strength (Gomez et al., 2002; Smith et al., 2006; Robertson et al., 2009; Jurado et al., 2010). AKAP5 is linked to NMDARs via PSD-95 (Colledge et al., 2000). AKAP5 is known to be a master scaffolding protein that links many proteins including kinases, phosphatases, cadherins, F-actin, MAGUKs and PIP_2_ together with ion channels and receptors to regulate activity dependent signaling processes at synapses (Tunquist et al., 2008; Sanderson and Dell’Acqua, 2011). Many of the proteins binding to AKAP5 (such as PKA, PP2B) also regulate GABA_B_Rs and perhaps there is possibility that AKAP5 scaffolding function may be required for glutamate/GABA receptors cross-talk.

Yotiao is another AKAP protein derived from alternative splicing of AKAP9 (also known as AKAP350/450) and plays a major role in regulating NMDARs. Yotiao was first identified as a binding partner of the GluN1 subunit and later found to be an AKAP via its ability to bind PKA-RII subunits *in vitro* (Lin et al., 1998; Westphal et al., 1999). Yotiao binds both protein phosphatase 1 (PP1) and PKA to form a phosphatase-kinase signaling complex with the GluN1A receptor splice variant. The Yotiao-PP1-PKA complex functions as dual switch, in that activation of anchored PKA enhances NMDAR currents while activation of PP1 exerts an inhibitory effect on NMDAR activity (Westphal et al., 1999; Colledge et al., 2000).

GABA_B1_Rs were previously shown to interact with a scaffold protein, GISP that enhances cell surface expression of heteromeric complex GABA_B1_/GABA_B2_ (Kantamneni et al., 2007). GISP is an AKAP9 C-terminal splice variant with more than 90% similarity to AKAP9 but lacking any RII domain, which are PKA binding sites (Kantamneni et al., 2007). As mentioned previously, the NMDAR binding protein Yotiao is also an AKAP9 splice variant, but within the N-terminal region. Therefore, theoretically, AKAP9 could interact simultaneously with NMDARs and GABA_B_Rs as well as regulatory protein kinases and phosphatases. Thus, while speculative, it is tempting to suggest that AKAP9 functions to assemble the signaling complex responsible for mediating the observed cross-talk between the NMDARs and GABA_B_Rs. From expression studies it is known that AKAP9 is expressed in the brain and localized to synapses (Collado-Hilly and Coquil, 2009). In similarity to the AKAP5-CaN_PP2B-PKA complex, the AKAP9-PKA-PP1 complex might exist as one large macromolecular complex held together with receptor proteins such as GABA_B_Rs and NMDARs. At least in yeast-two hybrid assay it has been confirmed that GISP does not interact with NMDAR sub-type 1 (Kantamneni et al., 2007). GISP binding to other subtypes of NMDARs or Yotiao binding to GABA_B_Rs has not been tested, and that this warrants further work. Another protein that may potentially mediate direct crosstalk between GABA_B_R signaling and glutamate receptor signaling is CaMKII. CaMKII is a Ca^2+^ calmodulin dependent protein kinase, previously been shown to interact with both GABA_B_ and NMDA receptors and regulate NMDAR mediated plasticity (Bayer et al., 2001; Guetg et al., 2010; El Gaamouch et al., 2012). Unlike the earlier examples of indirect receptor modulation, AKAPs and other signaling molecules like CaMKII potentially function as direct links between glutamate and GABA_B_ receptors. If further characterized these complexes may eventually serve as potential drug targets.

## Conclusions

Taken together we can conclude that there is very tight regulation between glutamate and GABA_B_ receptors. Regulation of NMDAR-mediated synaptic signals by GABA_B_Rs comprises a powerful mechanism for controlling the major excitatory systems in brain. Conversely, NMDAR-mediated control of GABA_B_Rs is clearly an important emerging concept in dictating the balance of excitability in the brain. Studying the trafficking and signaling pathways utilized by these excitatory and inhibitory receptors in an integrated manner will undoubtedly provide more understanding of these critical regulatory mechanisms and will ultimately shed light on how the balance between excitatory and inhibitory neurotransmission is dictated in the brain. While many examples of interactions between glutamate and GABA_B_ receptors have been discovered, importantly, the molecular players involved in mediating this cross-talk are only just beginning to be discovered. With this in mind, investigation of the potential players in these processes, such as the AKAPs, is an exciting future avenue of study. Ultimately, targeting these specific regulatory pathways may form the basis of new therapies to treat a number of neurological disorders that are characterized by aberrant balance between excitatory and inhibitory neurotransmitter systems in the brain.

## Conflict of Interest Statement

The author declares that the research was conducted in the absence of any commercial or financial relationships that could be construed as a potential conflict of interest.
